# Recruitment order of motor neurons promoted by epidural stimulation in individuals with spinal cord injury

**DOI:** 10.1152/japplphysiol.00293.2021

**Published:** 2021-08-12

**Authors:** Jaime Ibáñez, Claudia A. Angeli, Susan J. Harkema, Dario Farina, Enrico Rejc

**Affiliations:** ^1^Department of Bioengineering, Imperial College London, London, United Kingdom; ^2^Department of Clinical and Movement Neurosciences, Institute of Neurology, University College London, London, United Kingdom; ^3^Kentucky Spinal Cord Injury Research Center, University of Louisville, Louisville, Kentucky; ^4^Department of Bioengineering, University of Louisville, Louisville, Kentucky; ^5^Frazier Rehabilitation Institute, University of Louisville Health, Louisville, Kentucky; ^6^Department of Neurological Surgery, University of Louisville, Louisville, Kentucky

**Keywords:** epidural stimulation, motor neuron, spinal cord injury, standing, recruitment order

## Abstract

Spinal cord epidural stimulation (scES) combined with activity-based training can promote motor function recovery in individuals with motor complete spinal cord injury (SCI). The characteristics of motor neuron recruitment, which influence different aspects of motor control, are still unknown when motor function is promoted by scES. Here, we enrolled five individuals with chronic motor complete SCI implanted with an scES unit to study the recruitment order of motor neurons during standing enabled by scES. We recorded high-density electromyography (HD-EMG) signals on the vastus lateralis muscle and inferred the order of recruitment of motor neurons from the relation between amplitude and conduction velocity of the scES-evoked EMG responses along the muscle fibers. Conduction velocity of scES-evoked responses was modulated over time, whereas stimulation parameters and standing condition remained constant, with average values ranging between 3.0 ± 0.1 and 4.4 ± 0.3 m/s. We found that the human spinal circuitry receiving epidural stimulation can promote both orderly (according to motor neuron size) and inverse trends of motor neuron recruitment, and that the engagement of spinal networks promoting rhythmic activity may favor orderly recruitment trends. Conversely, the different recruitment trends did not appear to be related with time since injury or scES implant, nor to the ability to achieve independent knees extension, nor to the conduction velocity values. The proposed approach can be implemented to investigate the effects of stimulation parameters and training-induced neural plasticity on the characteristics of motor neuron recruitment order, contributing to improve mechanistic understanding and effectiveness of epidural stimulation-promoted motor recovery after SCI.

**NEW & NOTEWORTHY** After motor complete spinal cord injury, the human spinal cord receiving epidural stimulation can promote both orderly and inverse trends of motor neuron recruitment. The engagement of spinal networks involved in the generation of rhythmic activity seems to favor orderly recruitment trends.

## INTRODUCTION

Chronic, clinically motor complete spinal cord injury (SCI) disrupts the communication within the nervous system, leading to loss of motor function below the level of injury. This diagnosis is associated with a substantial decrease in quality of life (1) and severe limitations for neurological and functional recovery (2, 3). In the last decade, proof of principle studies have demonstrated that the application of spinal cord epidural stimulation (scES) combined with activity-based training can promote remarkable motor function recovery in this population as well as in individuals with incomplete SCI (4–8). These studies suggest that scES can modulate the excitability of the spinal circuitry controlling posture and locomotion, so that sensory information can serve as a source of control for generating appropriate motor patterns during standing and stepping. These and other studies (9–12) also showed that scES can re-enable volitional motor control of the paralyzed limbs during nonweight-bearing motor tasks as well as overground standing and stepping, conceivably via residual supraspinal connectivity to the lumbosacral spinal circuitry that is nondetectable and/or nonfunctional when scES is not provided.

Experimental and computational studies have enhanced our understanding of the mechanisms underlying scES-promoted motor function recovery after SCI, which is crucial to further improve the application of scES and progress toward functional recovery. To date, the prevailing view is that scES recruits dorsal root fibers carrying somatosensory signals, and particularly proprioceptive information (13, 14), at their entry into the spinal cord as well as along the longitudinal portions of the fiber trajectories (15–21). This leads to altering the excitability of spinal circuits to a level that can enable sensory information as well as residual supraspinal inputs to become sources of motor control (6, 9, 13, 22, 23), resulting in spinal motor neuron activation by engaging monosynaptic and polysynaptic circuits (14, 24). It is also recognized that stimulation parameters play a key role in determining the resultant activation pattern by determining the extent and characteristics of the modulation of the sensory-motor pathways impacted by scES (13, 21, 25–27).

Interestingly, there is still an important gap in knowledge related to how spinal motor neurons are recruited when the activation pattern is promoted by epidural stimulation after SCI. In able-bodied individuals, voluntary muscle contractions of increasing levels are accompanied by an orderly recruitment of motor neurons according to their size, with smaller neurons being recruited before larger ones (28). The orderly recruitment order has an impact on limiting muscle fatigue as fast, fatigable motor units are recruited after slow, fatigue-resistant motor units. In several applications of electrical stimulation of the motor nerve fibers (e.g., neuromuscular electrical stimulation), the recruitment order can be random and nonselective (29, 30) or reversed (31), leading to exaggerated metabolic cost of muscle contractions and rapid onset of muscle fatigue, which substantially limit their potential as an assistive technology (32, 33). Although speculative arguments have been proposed for a more physiological motor neuron recruitment promoted by scES as compared with other electrical stimulation techniques (34), no attempts to investigate scES-promoted motor neuron recruitment have been reported yet. However, this aspect of scES is important to contribute to understand the potential of this neuromodulation technology for functional restoration.

The recruitment of motor neurons can be investigated by assessing the characteristics of the activated muscle fibers. The size of motor neurons is associated with the size of their axons and to the diameter of the innervated muscle fibers (28). This implies that larger motor neurons innervate muscle fibers with larger diameters. Because the diameter of muscle fibers is proportional to the propagation velocity of action potentials along the fibers, muscle fiber conduction velocity is effectively a size principle parameter (35), equivalent to the soma size of the motor neurons (36). Estimates of muscle fiber conduction velocity in relation to the amplitude of muscle activity are therefore an indirect indicator of the recruitment order of the activated motor neurons (37–40). In clinical settings, muscle fiber conduction velocity can be reliably measured using arrays of sensors measuring the conduction of electrical fields along the muscles (41).

In the present study, we aimed at assessing whether scES, which provides input to motor neurons via afferent pathways, promotes orderly, inverse, or nonselective motor neuron recruitment trends during standing in individuals with chronic motor complete SCI. To do so, we analyzed the relationship between propagation velocity along the muscle fibers and amplitude of the EMG responses evoked by scES.

## MATERIALS AND METHODS

### Participants

Five individuals with chronic, clinically motor complete and sensory complete or incomplete SCI were included in this study (Table 1). Research participants signed an informed consent for lumbosacral spinal cord epidural stimulator implantation, stimulation, activity-based training, and physiological monitoring studies, which were conducted according to the standards set by the Declaration of Helsinki and approved by the University of Louisville Institutional Review Board. Before epidural stimulator implantation, the International Standards for Neurological Classification of Spinal Cord Injury (42) was used to classify each injury using the ASIA (American Spinal Injury Association) Impairment Scale (AIS). Research participants had been enrolled in interventional studies focused on facilitating standing (all individuals), stepping (B07 and A45), lower limb volitional movements (B24, A100, B07, and A45), and/or recovery of cardiovascular function (A105) before participating in this study. Characteristics of these interventions have been reported in previous publications (4, 9, 43, 44).

**Table 1.** Characteristics of the research participants

Pub ID	Gender	Age, yr	Time between Injury and Surgery, yr	Injury Level	AIS	Time Since scES Implant, yr
B24	M	25.5	6.7	C6	B	0.6
A100	M	52.0	16.6	C4	A	0.4
B07	M	24.0	3.4	T2	B	9.9
A45	M	24.2	2.2	T4	A	8.2
A105	M	33.7	10.0	C4	A	0.7

Injury level is the neurological level of the lesion by AIS (American Spinal Injury Association (ASIA) Impairment Scale; (42). Pub ID, publication identifier.

### Spinal Cord Epidural Stimulation Implant

During the scES implantation procedure, a midline bilateral laminotomy was performed typically at the L1-L2 disk space. An electrode array with 16 contacts (Medtronic Specify 5–6-5 lead, size: 10 mm × 64.2 mm; contact size: 1.5 mm × 4.0 mm; edge-to-edge contact spacing: 4.5 mm–column–and 1.0 mm–row) was placed into the epidural space at midline. Electrophysiological mapping was performed after initial placement to optimize the location of the paddle electrode based on evoked responses recorded from bilateral surface EMG electrodes (Motion Lab Systems, Baton Rouge, LA) placed over representative lower limb muscles. After the final placement of the electrode array, the electrode lead was tunneled subcutaneously and connected to the neurostimulator (Medtronics, Intellis in participants B24, A100, B07, and A105; RestoreADVANCED in participant A45). The neurostimulator delivered monophasic, rectangular pulses.

### Experimental Procedures

The goal of this study was to characterize the motor neuron recruitment characteristics promoted by scES during standing. In particular, we focused this analysis on the vastus lateralis (VL) muscle because it is one of the primary antigravity muscles activated during standing in people with motor complete SCI receiving scES. Standing experimental sessions were performed overground without body weight support, using either a custom-designed standing apparatus that comprises horizontal bars anterior and lateral to the individual, which were used for upper extremity support (6) (participants A100 and A105), a walker that was fixed to a wider aluminum frame base (B24), or a regular walker (B07 and A45). scES was applied while the participant was seated. The sit to stand transition was performed with research participants using their upper limbs to partially pull themselves into a standing position, and trainers positioned at the pelvis and knees manually assisting as needed the transition. If needed, participants with limited upper limb function were also assisted by trainers at the axillary triangle during the sit to stand transition. When a stable standing position was achieved, if the knees, hips or trunk flexed beyond the normal standing posture, manual external assistance was provided at the knees distal to the patella to promote extension, at the hips below the iliac crest to promote hip extension and anterior tilt, and at the axillary triangle to promote trunk extension. Research participants self-assisted balance control using their upper limbs during the standing events considered for this study. Seated resting periods occurred when requested by the individuals.

### Stimulation Parameters and Approach

**Figure 1.**
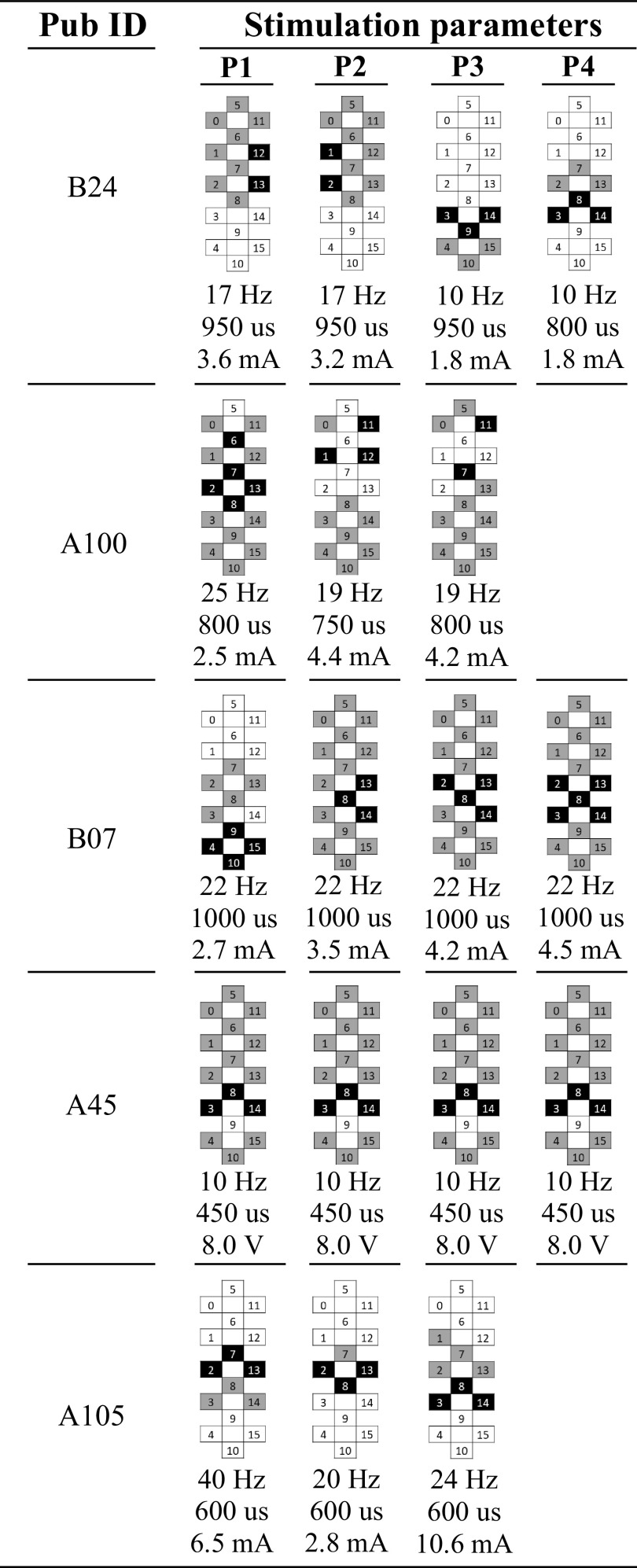
Epidural stimulation parameters applied to facilitate standing. Multiple stimulation programs (P1 to P4) were concurrently applied to the research participants to facilitate standing during the recordings of high-density electromyography. Multiple stimulation programs were delivered in an interleaved fashion (A45), or with independent frequencies (B24, A100, B07, and A105), depending on the stimulator unit implanted (Medtronics RestoreADVANCED or Intellis, respectively). Stimulation frequency, pulse width, amplitude, and electrode configuration (cathodes in black, anodes in gray, and inactive in white) are reported for each stimulation program.

Tonic scES with individual-specific parameters, which remained fixed throughout the high-density EMG recordings, was applied to facilitate standing. These parameters are reported in Fig. 1.

Briefly, stimulation parameters were selected to promote the modulation of the spinal circuitry excitability so that peripheral sensory information, and possibly residual supraspinal inputs, could be used as sources of motor control to generate lower limb activation patterns effective for standing (6, 7, 25, 45). This approach results in the application of near-motor threshold stimulation amplitudes that directly elicits negligible EMG activity and no leg movements in sitting. However, without any change in stimulation parameters, peripheral sensory information (e.g., loading of the legs and lower limb extension associated to the sit-to-stand transition) is integrated by the spinal circuitry to result in the generation of robust lower limb activation patterns. Generally, one of the primary components of the standing activation pattern are scES-evoked responses, which show relevant amplitude and shape modulation, and may or may not be linked to the stimulation pulses delivered to the spinal cord (25, 45).

### Selection of Stimulation Parameters

The process of selection of scES parameters for standing had been already carried out for all participants before the present study because all individuals had already practiced standing as part of other interventional studies (ClinicalTrials.gov identifiers NCT02339233 or NCT03364660). Briefly, dedicated guidelines (25) contributed to the initial selection of subset of stimulation parameters to be tested in standing, which were then finalized through an evidence-based approach during two or three experimental sessions. These parameters were also refined as needed throughout stand training interventions, following the same guidelines (25).

The subset of electrode configurations tested to facilitate standing were primarily identified based on three factors: *1*) individualized maps of motor pools activation, which were determined during spatiotemporal mapping assessments performed ∼2–3 wk after the surgical implantation of the scES unit. These assessments considered, among others, muscle activation responses to different localized two-electrode configurations, using 2 Hz stimulation frequency and increasing amplitude, with the research participants relaxed in supine position. Stimulation amplitude and evoked potentials peak-to-peak amplitude for each electrode configuration tested were then reported as colormaps for each investigated muscle (46). This information was used to adjust the position of cathodes in order to target primarily extensors muscle groups while limiting the activation of primary flexor muscles. *2*) Earlier evidence of lower limb extension pattern generation. Previous literature suggested that cathodes positioned in the caudal area of the lumbosacral spinal cord, and more caudal than the anodes, can promote better motor pattern characteristics of standing behavior (20, 26, 47). *3*) Topographical organization of the activation pattern, which reflects the preferred activation of proximal or distal muscle groups by focusing the electrode field caudally or rostrally, respectively (15). Also, in case of activation differences between left and right lower limb, active electrodes can be unbalanced between lateral columns of the electrode array to compensate these differences (21). The application of multiple (up to 4) stimulation programs was also explored to access more specific locations of the spinal circuitry and facilitate the activation of specific muscle groups (7).

Earlier studies showed that high scES amplitude applied to SCI individuals in supine position can directly elicit lower limb tonic extension or locomotor-like patterns, depending of the stimulation frequency applied (i.e., 5–15 Hz or 25–60 Hz, respectively) (26, 48). However, we initially applied near-motor threshold stimulation amplitudes, which did not elicit directly lower limb movements in sitting, at higher stimulation frequency (i.e., 25 Hz) to favor the integration of afferent input and residual supraspinal input through the greater involvement of interneurons (49, 50), and a more physiological (i.e., nonpulsatile) muscle contraction (25). Stimulation frequency and amplitude were then modulated synergistically during standing to identify the highest stimulation frequency that promoted an EMG pattern effective to bear body weight. This is consistent with our approach of using scES for enabling the spinal circuitry to integrate weight bearing-standing related sensory information, and possibly residual descending input, to generate motor patterns effective for standing, rather than using scES to drive an activation pattern.

### Data Acquisition

To assess the velocity of the scES-evoked responses traveling along the VL muscle fibers, high-density EMG (HD-EMG) recordings were acquired by a multichannel EMG amplifier (Quattrocento, OT Bioelettronica, Torino, Italy) and rectangular HD-EMG grids (5 columns and 13 rows; gold coated; 1-mm diameter; 8-mm interelectrode distance) (51). Before placing the grids, the skin was shaved, lightly abraded, and cleansed with a 70% alcohol solution. HD-EMG signals were acquired in monopolar mode. The grid center was positioned over the VL muscle belly, ensuring an optimal coverage of the muscle. Together with the HD-EMG recordings, a digital manual pulse signal was acquired to define experimental event markers.

### Estimation of Motor Neuron Recruitment Order from HD-EMG

To infer the motor neuron recruitment order during standing with scES, we analyzed the changes in conduction velocity of scES-evoked compound responses collected from the VL muscle as a function of motor unit recruitment. Specifically, we assessed the relation between the amplitude of the evoked responses and their conduction velocity along the muscle fibers. This analysis relies on the consideration that progressive increases in the amplitude of the evoked muscle responses are associated with the recruitment of new motor units (regardless of their size), and that progressive decreases of the evoked responses’ amplitude are associated with the derecruitment of motor units. This allowed us to infer that when conduction velocities and amplitudes of evoked muscle responses were positively correlated, motor units of progressively higher conduction velocity were recruited (orderly recruitment trend), whereas a negative correlation indicated a progressive recruitment of motor units with decreasing values of conduction velocity (inverse recruitment trend; 40).

In offline analysis, the HD-EMG signals were band-pass filtered (20–500 Hz band, 2nd order zero-lag Butterworth filter) and the optimal column and rows of the recording grids for the estimation of conduction velocities were determined by visual inspection. In particular, we initially performed a manual selection of the column in which the largest number of recording channels showed clear muscle activation patterns above the background noise level. Then, channels in the selected grid column showing unidirectional propagation pattern of the scES-evoked responses were selected. The focus on EMG signals derived from only one side of the innervation zone ensured a robust performance of the automatic algorithm used to estimate conduction velocity along the muscle fibers. Double differential signals were then obtained from the monopolar recordings in the selected channels. The application of this spatial filter enhanced the part of the recorded signals that showed a traveling pattern across consecutive channels (i.e., motor unit action potentials) while it reduced stimulation artifacts and other distant sources contributing to the EMG signals recorded (52, 53). Muscle fiber conduction velocity and mean absolute amplitude were subsequently obtained in sliding windows of 30 ms, which was selected based on the maximum lengths of the EMG evoked responses observed for the different participants, with a stepping of 4 ms between consecutive windows. For each research participant, we considered for analysis up to 100 s of the best stable standing bout with consistent external assistance defined as the longest stable standing bout performed with the greater amount of body segments (knees, hips, and trunk) controlled independently.

The muscle fiber conduction velocity was obtained with a multichannel maximum-likelihood algorithm that has previously shown to estimate velocities with an associated standard deviation below 0.1 m/s (37). The mean absolute amplitude for each 30-ms window of analysis was obtained by averaging the amplitudes of the rectified EMG signals from the channels used to estimate the conduction velocity. The resulting mean absolute amplitude signal was used to determine the time points at which scES-evoked responses were located. In particular, the amplitude peaks were automatically detected by setting a threshold for peak detection at 20 µV. The mean absolute amplitude and muscle fiber conduction velocity pairs of values detected at each mean absolute amplitude peak time were considered for further analysis. In particular, the relationship between the two variables was investigated by: *1*) Mann–Kendall test, to assess whether a statistically significant trend was present in muscle fiber conduction velocity as a function of mean absolute amplitude; *2*) Spearman correlation, to quantify strength, direction, and statistical significance of the association between these two variables. The slope of the linear regression between muscle fiber conduction velocity and mean absolute amplitude data points was also assessed. In addition, the variability of the overall EMG pattern was quantified by the coefficient of variation of the EMG linear envelope obtained by filtering the rectified EMG signals through a low-pass filter (fc = 4 Hz, Butterworth zero-phase filter, order 3) (45).

## RESULTS

### scES-Evoked Responses Can Be Characterized by High-density-EMG

**Figure 2.**
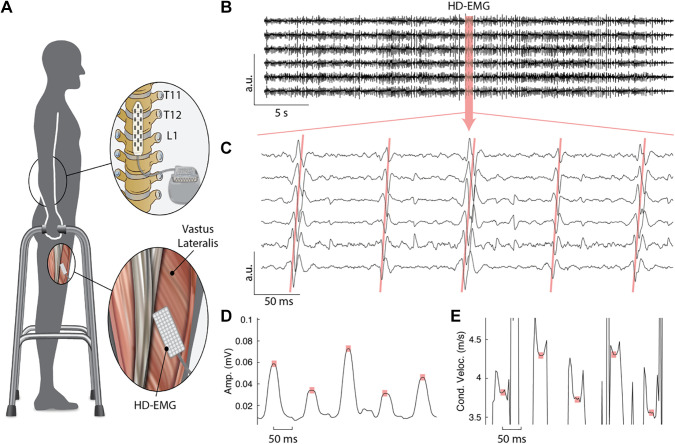
Experimental setup. *A*: exemplary schematics of a research participant implanted with a spinal cord epidural stimulation (scES) unit standing overground with an assistive device for balance control (i.e. walker) while receiving scES to promote standing. High-density electromyography (HD-EMG) was collected from the vastus lateralis muscle. *B*: EMG recordings from the optimal set of channels (those allowing a reliable estimate of propagation velocities) along a column of the recording electrodes grid. *C*: enlarged view of the EMG signals demonstrating propagation of the scES-evoked responses. *D* and *E*: amplitude and conduction velocity of the evoked responses showed in *C*, assessed using a 30 ms sliding window (see details in material and methods). Red squares identify the time points associated with peak amplitude of scES-evoked responses, which are considered for further analysis. Stimulation parameters are reported in Fig. 1, research participant A100.

EMG collected via surface high-density-matrix placed on the VL muscle during standing enabled by scES showed evoked responses that demonstrated clear propagation patterns along the fiber length (Fig. 2, *A*–*C*). This allowed the reliable assessment of their amplitude and conduction velocity along the muscle fiber (Fig. 2, *D* and *E*).

### Motor Neuron Recruitment Pattern during Standing with scES

**Figure 3.**
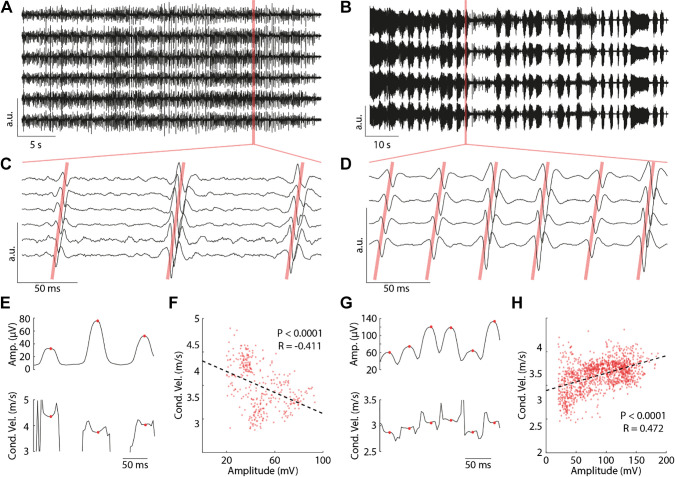
Data collected from research participant B24 (*left*) and A45 (*right*) during standing with spinal cord epidural stimulation (scES). *A* and *B*: raw vastus lateralis EMG data collected from the selected electrode grid channels to estimate amplitude and conduction velocity of the scES-evoked responses. *C* and *D*: enlarged view of the EMG signals demonstrating propagation of the scES-evoked responses along the muscle fibers, as exemplified by the diagonal pink lines. *E* and *G*: Amplitude (*top*) and conduction velocity (*bottom*) of the evoked responses reported in *C* and *D*. *F* and *H*: conduction velocity values are plotted as a function of the corresponding scES-evoked response amplitudes. Stimulation parameters are reported in Fig 1.

The integration of tonic scES applied to facilitate standing with peripheral sensory information and residual supraspinal inputs enabled the spinal circuitry to generate overall continuous lower limb muscle activation patterns (Fig. 3*A*) or activation patterns characterized by the presence of EMG bursts (Fig. 3*B*). Importantly, the propagation pattern of scES-evoked responses was appropriately assessed for both types of activation (Fig. 3, *C* and *D*). Average muscle fiber conduction velocity of the scES-evoked responses detected during standing was similar in three of the five individuals of this study, ranging between 4.0 and 4.3 m/s (Table 2). The other two individuals showed lower average conduction velocity values (3.0 m/s and 3.2 m/s, Table 2).

**Table 2.** Characteristics of the standing bout examined and EMG activity collected from the vastus lateralis muscle during standing with spinal cord epidural stimulation

Pub ID	Standing Condition	Duration, s	Amplitude, mV	Cond Vel, m/s	MK *P*-Value	Spear *R*	Spear *P* Value	Slope m·s^−1^·mV^−1^	Coeff Var
B24	H_a K_i	41	48.8 ± 17.2	4.0 ± 0.3	<0.0001	−0.411	<0.0001	−8.2	0.26
A100	H_a K_a	100	47.1 ± 22.5	4.4 ± 0.3	<0.0001	0.155	0.001	2.3	0.92
B07	H_i K_i	60	49.3 ± 21.8	4.1 ± 0.2	<0.0001	−0.181	<0.0001	−1.9	0.29
A45	H_a K_i	90	89.5 ± 38.9	3.2 ± 0.2	<0.0001	0.472	<0.0001	2.6	0.70
A105	H_a K_i	39	25.1 ± 0.2	3.0 ± 0.1	<0.0001	−0.581	<0.0001	−26.7	0.37

Standing condition defining manual assistance (a) or independent extension (i) of the hips (H) or knees (K). Duration, duration of the standing bout. MK, Mann–Kendall test to assess the statistical significance of the trend present in conduction velocity (Cond Vel) as a function of amplitude of evoked responses. Spear. Spearman correlation. Slope, slope of the linear regression between conduction velocity and amplitude of evoked responses. Coeff var, coefficient of variation of the EMG linear envelope to quantify the variability of the EMG pattern. Pub ID, publication identifier.

Amplitude and conduction velocity of scES-evoked responses were modulated over time (Fig. 2, *D* and *E* and Fig. 3, *E* and *G*), while stimulation parameters (Fig. 1) and standing condition (Table 2) remained constant. Variations in amplitude of scES-evoked responses were associated to changes in conduction velocity that were individual-specific. In some participants (e.g., B24, Fig. 3*E*), an increase in amplitude corresponded to a decrease in conduction velocity, which indicated an inverse recruitment order. In other individuals (e.g., A45, Fig. 3*G*), the trend of conduction velocity as a function of amplitude was in agreement with an orderly recruitment. These opposite trends resulted in a significant inverse linear relationship (*r* = −0.411; *P* < 0.0001) for participant B24 (Fig. 3*F*), whereas a significant direct relationship (*r* = 0.472; *P* < 0.0001) was found for A45 (Fig. 3*H*).

We observed statistically significant trends of conduction velocity as function of the amplitude of the scES-evoked responses in all five individuals of this study (Mann–Kendall tests indicated significant trends with *P* < 0.0001; Table 2). These significant linear trends were inverse for participants B24, B07, and A105, whereas they were direct for the other two individuals (Table 2). Interestingly, these two direct trends were associated with EMG patterns characterized by the presence of bursting activity (e.g., Fig. 3*B*). Conversely, the inverse relationship between conduction velocity and amplitude of scES-evoked potentials was associated with overall continuous EMG patterns (e.g., Fig. 3*A*). To quantify these two different EMG patterns promoted by scES (i.e., presence of bursts vs. overall continuous), we determined the variability of the overall EMG pattern by computing the coefficient of variation of the EMG linear envelope. A high coefficient of variation of the EMG envelope indicates the presence of bursting EMG activity while a lower coefficient of variation indicates overall continuous activity. Table 2 shows that a direct relation between conduction velocity and amplitude of the evoked EMG responses was observed only for the two participants who demonstrated high coefficient of variation of the EMG pattern, suggesting that bursting activity could reflect the orderly recruitment order of motor neurons.

Finally, the two different motor neuron recruitment trends were not clearly related to the research participants’ characteristics (i.e., time since injury or scES implant; Table 1), nor to their standing condition (i.e., independent or assisted knee extension) or muscle fiber conduction velocity values (Table 2).

## DISCUSSION

We studied the recruitment order of motor neurons during standing motor function enabled by scES in individuals with chronic motor complete SCI. The proposed approach allowed the measurements of propagation velocity of the action potentials generated by motor neuron activity in the innervated muscle fibers resulting in scES-evoked responses. We found that conduction velocity and amplitude of scES-evoked responses were modulated over time, and that this modulation resulted in significant trends of inverse or orderly motor neuron recruitment order.

scES optimized for standing is applied at near-motor threshold amplitudes in sitting, eliciting little or negligible EMG activity, and no movement (7, 25, 45). This stimulation approach did not directly induce motor pool activations appropriate for standing. Rather, it modulated the excitability of the lumbosacral spinal circuitry controlling posture so that the integration of afferent inputs related to the sit to stand transition and loading of the legs, and eventual residual descending inputs, served as a source of control for generating activation patterns effective for standing (6, 7, 25). However, computational and experimental studies also suggested that tonic scES delivered at higher frequencies can interfere with the natural flow of proprioceptive information to the brain and spinal cord in humans, because of the propagation of antidromic signals delivered by the epidural stimulator (54). scES-evoked responses (Fig. 2, *B* and *C* and Fig. 3, *A*–*D*) generally characterize the standing activation patterns enabled by spinal cord stimulation (25, 45, 55). In the present study, they were promoted by the complex interaction of multiple stimulation programs for each individual (P1 to P4, Fig. 1). These stimulation programs were delivered in an interleaved fashion (A45), or with independent frequencies (B24, A100, B07, and A105), depending on the stimulator unit characteristics. Conduction velocity and amplitude of the scES-evoked responses were modulated over time (Fig. 2, *D* and *E* and Fig. 3, *E* and *G*) and presented a wide range of values (Fig. 3, *F* and *H*) whereas stimulation parameters and standing condition remained constant. This supports the perspective of a dynamic involvement of the spinal circuitry leading to the activation of variable subsets of motor neurons to result in the different scES-evoked response characteristics. A concurrent or alternative mechanistic hypothesis is that the overall spinal cord excitability promoted by scES and residual inputs to the spinal circuitry was close to the excitability threshold required to activate a subpopulation of motor neurons, which were therefore activated inconsistently.

The range of conduction velocity values detected in the present study (Table 2; Fig. 3) is consistent with the values reported for the VL muscle of able-bodied individuals during isometric ramp contractions at different effort levels (56, 57). Similar assessments of isometric ramp contractions in able-bodied subjects (40) have also demonstrated occasional individual-specific trends of conduction velocity that are consistent with the different conduction velocity values herein observed for A105 and A45 with respect to the other three participants. The SCI population presents interindividual differences in the neuromuscular system, which can be due to the characteristics of the injury itself, those of the spinal circuitry reorganization after injury, and pharmacological treatments, among others (58, 59). SCI also results in muscle atrophy and in a shift toward fast-fatigable muscle phenotype (60, 61). However, individual-specific characteristics such as muscle tone level (i.e., spasticity) can influence SCI-mediated skeletal muscle adaptations (62, 63). Importantly, SCI-induced muscle atrophy appears to be primarily due to a loss of muscle fibers, as the cross-sectional area of single muscle fibers was found similar between able-bodied individuals and chronic SCI individuals for all fiber types (60). These and others SCI-induced neuromuscular adaptations cannot be controlled in human research participants, and may account for the individual-specific slope and strength of the “conduction velocity versus amplitude” linear regressions (Table 2). Nevertheless, the findings herein reported support the concept that, after motor complete SCI, the human spinal cord receiving epidural stimulation can promote both orderly and inverse trends of motor neuron recruitment order. It is worth noting that the different recruitment trends did not appear to be related with time since injury or scES implant (Table 1), nor to the ability to achieve independent knees extension, nor to the muscle fiber conduction velocity values (Table 2).

Limited literature (64, 65) based on the assessment of force time-to-peak during twitch muscle contractions generated by soleus H-reflex proposed that synaptic Ia input may lead to motor neuron recruitment in an orderly fashion (smallest to largest) according to the Henneman size principle (28). Here, we have assessed motor neurons recruitment order by investigating the relation between amplitude of the muscle electrical activity and conduction velocity of muscle signals along the fibers (35). A positive slope of this association suggests orderly recruitment trends by size, whereas a negative slope points toward an inverse recruitment trend (39). Our data suggest that scES, which recruits primarily large sensory fibers (e.g., Ia) to modulate the excitability of lumbosacral spinal circuitry (13, 14, 16, 20, 21), can promote both inverse and orderly trends of motor neurons recruitment order when applied to facilitate standing in individuals with motor complete SCI (Fig. 3; Table 2). Interestingly, moderate or weak direct trends of motor neuron recruitment were observed when EMG activity demonstrated bursting patterns (Fig. 3*B*; Table 2, participants A100 and A45). Conversely, moderate or weak inverse trends of motor neuron recruitment were observed when EMG pattern was overall continuous (Fig. 2*A*; Table 2, participants B24, B07, and A105). Mean and standard deviation values of conduction velocity and amplitude of scES-evoked responses were not univocally affected by the presence of bursting in the EMG pattern. For example, mean and standard deviation values of amplitude and conduction velocity observed for participant A100 (bursting pattern) are comparable to those assessed for B24 and B07 (overall continuous pattern; Table 2). This suggests that similar ranges of recruitment across activity levels and motor unit types were assessed for both EMG patterns in these individuals, and that the difference in motor neuron recruitment trends was not solely due to the fact that the bursting EMG pattern resulted in wider range of motor neuron recruitment.

The presence of EMG bursts during standing (i.e., when rhythmic afferent inputs from lower limbs were not provided) suggests that scES, together with standing-related sensory information and residual supraspinal inputs, engaged part of the spinal networks responsible for generating rhythmic activity (26, 66), which was not involved in the three individuals demonstrating overall continuous EMG patterns. In this case, the stimulation parameters applied interacted with the spinal circuitry in a suboptimal manner with respect to the motor task facilitated, because activation patterns containing EMG bursts were promoted during standing. Higher stimulation frequencies (e.g., 25–60 Hz) can facilitate the generation of rhythmic activity (26, 48). However, other factors such as electrode configuration (25), location of spinal cord stimulation and stimulation amplitude (24, 48) play a role in the characteristics of activation pattern generation. Nevertheless, the assessment of these activation patterns revealed that the engagement of different subset of neural networks within the spinal circuitry controlling posture and locomotion may favor different trends of motor neuron recruitment. Also, mechanisms of presynaptic inhibition can influence afferent inputs to the motor neurons (64, 67), and might contribute to the modulation of their recruitment characteristics (68). Presynaptic inhibition is modulated by spinal cord stimulation after SCI (69), and could therefore be another contributing mechanism to the characteristics of motor neuron recruitment in the population of the present study.

In conclusion, we characterized for the first time the recruitment order of motor neurons during standing enabled by epidural stimulation in individuals with motor complete SCI by measuring muscle fiber conduction velocity and amplitude of scES-evoked responses. We found that, after motor complete SCI, the human spinal circuitry receiving epidural stimulation can promote both orderly and inverse trends of motor neuron recruitment order. The proposed approach can allow to investigate the effects of different factors, such as stimulation parameters and training-induced neural plasticity, on the characteristics of motor neuron recruitment order. This proposed approach could be also implemented in real-time, contributing to closed-loop systems devoted to the selection of stimulation parameters. These future directions could contribute to improve the mechanistic understanding and effectiveness of epidural stimulation-promoted motor function recovery after severe SCI.

## GRANTS

This work was supported by Christopher and Dana Reeve Foundation, Leona M. & Harry B. Helmsley Charitable Trust, Kessler Foundation, National Institutes of Health (1R01EB007615), UofL Health - University of Louisville Hospital, and Medtronic Plc.

## DISCLOSURES

No conflicts of interest, financial or otherwise, are declared by the authors.

## AUTHOR CONTRIBUTIONS

J.I., S.J.H., D.F., and E.R. conceived and designed research; J.I., C.A.A., and E.R. performed experiments; J.I., C.A.A., and E.R. analyzed data; J.I., D.F., and E.R. interpreted results of experiments; J.I. and E.R. prepared figures; J.I., D.F., and E.R. drafted manuscript; J.I., C.A.A., S.J.H., D.F., and E.R. edited and revised manuscript; J.I., C.A.A., S.J.H., D.F., and E.R. approved final version of manuscript.

## DATA AVAILABILITY

Data that support the findings and software routines developed for data analysis will be made available through material transfer agreement upon reasonable request.
